# Moderately strong intraclass correlations between actigraphic and polysomnographic total sleep time and sleep efficiency in older adults with sleep disturbance

**DOI:** 10.1007/s11325-025-03326-y

**Published:** 2025-04-15

**Authors:** Matthew M. Rahimi, Craig L. Phillips, Nathaniel S. Marshall, Rick Wassing, Teha Pun, Ron R. Grunstein, Christopher J. Gordon

**Affiliations:** 1 CIRUS Centre for Sleep and Chronobiology, Woolcock Institute of Medical Research, Macquarie University, Sydney, NSW Australia; 2 Department of Health Sciences, Faculty of Medicine, Health and Human Sciences, Macquarie University, Sydney, Australia; 3 Macquarie Medical School, Faculty of Medicine, Health and Human Sciences, Macquarie University, Sydney, NSW Australia; 4 School of Psychological Sciences, Faculty of Medicine, Health and Human Sciences, Macquarie University, Sydney, NSW Australia; 5 Charles Perkins Centre Clinic, Royal Prince Alfred Hospital, Sydney, NSW Australia; 6 Faculty of Medicine and Health, University of Sydney, Sydney, NSW Australia

**Keywords:** Actigraphy, Older people, Polysomnography, Repeated measures, Sleep, GeneActiv

## Abstract

**Objective:**

To evaluate the reliability of the GeneActiv actigraphy device in measuring sleep parameters and compare its performance with polysomnography (PSG) in older adults with self-reported sleep disturbances.

**Methods:**

This sub-study was part of a pilot double-blinded randomized controlled crossover trial (CleverLights Study, ANZCTR ID 12619000138189). Participants (*n* = 12, mean age 67.7 years) underwent two nights of sleep studies with simultaneous GeneActiv actigraphy and PSG, separated by a 2-week interval. Sleep parameters including time in bed (TIB), total sleep time (TST), wake after sleep onset (WASO), sleep onset latency (SOL), sleep efficiency (SE), and number of awakenings were assessed. Intraclass Correlation Coefficients (ICCs) and Bland-Altman plots were used to determine reliability and agreement between methods.

**Results:**

GeneActiv actigraphy demonstrated strong correlations with PSG for TST (ICC = 0.79, *p* = 0.001) and SE (ICC = 0.85, *p* < 0.001), but tended to overestimate these parameters. Actigraphy also significantly underestimated the number of awakenings (ICC = 0.45, *p* = 0.021). Correlations with observed TIB (ICC = 0.30, *p* = 0.433), WASO (ICC = 0.33, *p* = 0.386), and SOL (ICC = 0.32, *p* = 0.056) were non-significant. Bland-Altman plots revealed proportional bias, especially in SOL and the number of awakenings.

**Conclusion:**

Compared to PSG, the GeneActiv actigraphy device provides reliable measurements for total sleep time and sleep efficiency, but agreement was weaker for wake after sleep onset, sleep onset latency, and the number of awakenings. The device showed consistent performance across multiple nights, suggesting good reproducibility. However, it systematically overestimated total sleep time and underestimates wake-related parameters, hence it may not fully replace PSG for detailed sleep assessments.

## Introduction

Accurate objective measurement of sleep parameters is crucial for understanding sleep quantity and quality. Polysomnography (PSG) is the gold standard for sleep measurement, providing detailed information about sleep stages, and physiological variables, such as heart and respiratory rates and eye and muscle movements. PSG typically requires an overnight stay in a sleep laboratory, which is expensive, time-consuming, and can be uncomfortable for patients. Actigraphy, which uses a wrist-worn accelerometer to measure sleep-wake patterns, has been shown to overestimate total sleep time (TST) and wake after sleep onset (WASO) [1, 2]. Among the various actigraphy devices, the GeneActiv (ActivInsight Inc, United Kingdom), has gained attention for its high level of accuracy and sensitivity in detecting sleep parameters [3]. GeneActiv features a tri-axis accelerometer, offering high-resolution 50 Hz data collection, similar to other research-grade devices such as Actiwatch [4]. However, it differs in its open-source software compatibility and ability to measure additional parameters like light exposure. Studies suggest GeneActiv’s sensitivity in sleep-wake detection rivals Actiwatch, but its reliability in specific populations, such as older adults, requires further exploration. Furthermore, there is limited research involving multiple nights of both PSG and actigraphy measurements in older adults, which may exhibit inconsistent sleep patterns and characteristics across nights. Previous research highlights challenges in using actigraphy in older populations due to increased nocturnal awakenings, decreased sleep efficiency, and fragmented sleep patterns [5, 6]. While GeneActiv has been validated in younger adults, studies evaluating its performance in older adults remain scarce [2].

We aimed to assess the reliability between sleep parameters measured by actigraphy, using the GeneActiv device, and in-lab PSG in older adults with reported sleep disturbance. Additionally, we aimed to evaluate the consistency of these reliability measures across individual nights to explore potential night-to-night variability in sleep assessment.

## Method

This was a sub-study of a pilot double-blinded randomized controlled crossover trial that aimed to investigate the effects of two different light therapy interventions on objectively measured sleep quality in people over 50 years who self-reported disturbed sleep (CleverLights Study: Clinical Trial registration ID 12619000138189 at ANZCTR.org.au; See clinical trial registry for inclusion/exclusion criteria.). The present analyses do not report the original hypotheses being tested in the CleverLights trial.

Between February 2021 and June 2022, participants completed two 3-night stays at the Woolcock Institute of Medical Research in Sydney, Australia, with 2 weeks washout period. During each visit, participants stayed in the laboratory for three nights: two for habituation and one for testing. PSG and actigraphy measurements were recorded on the third night. Throughout their stay, participants maintained their habitual bedtime and wake time, as determined from a 7-day sleep diary. Sleep patterns were monitored using GeneActiv actigraphy, which recorded tri-axis accelerometry data at 50 Hz. Additionally, participants’ electrophysiological brain activity during sleep was recorded using high-density electroencephalography (HD-EEG) (Electrical Geodesics (EGI, Inc), United States).

Sleep was staged by a single experienced polysomnographic technologist and sleep parameters were calculated following standard guidelines by the American Academy of Sleep Medicine (AASM) criteria [7]. The tri-axis actigraphy data were transformed into the Euclidean Norm Minus One [8] and the angle of the orientation of the device in the z-axis [9], averaged across 5 s epochs, and scored through a custom open-source software (i.e., Cicada, https://cicada-actigraphy-suite.readthedocs.io). Sleep parameters were calculated as follows. The time in bed (TIB) was determined manually using the Euclidean Norm Minus One, sleep diaries and lab notes. Within the TIB period, epochs were annotated as sustained inactive where the change in the angle of the actigraph was less than 5 degrees for at least 5 min. The sleep period was the period between the first and last sustained inactivity epoch. Sleep onset latency (SOL) was the time between the onset of the TIB period to the first epoch of sustained inactivity, wake after sleep onset (WASO) was the total duration of non-sustained inactivity epochs within the sleep period, total sleep time (TST) was the total duration of all sustained inactivity epochs, sleep efficiency (SE) was calculated as 100 × (TST / TIB), and number of awakenings were the total number of non-sustained activity bouts within the sleep period.

### Analysis

Descriptive statistics were utilised to characterise the study sample and evaluate data distributions. All descriptive statistics are presented as mean ± SD unless otherwise specified. Data analysis was conducted using SPSS (version 26). Data was visually inspected to confirm normality. We used the Intraclass Correlation Coefficient (ICC) to evaluate the reliability i.e., the level of absolute agreement for both average measures across two nights and separately for each night between the two devices. Additionally, Pearson correlation (Spearman rho for non-normally distributed data) was employed to investigate the relationship between between PSG measurements and actigraphy measurements.

Bland-Altman plots [10] were constructed to visually display the agreement between actigraphy and PSG sleep parameters. These plots allowed for the examination of proportional bias, which indicates that the difference between measurements from PSG and actigraphy varies systematically as a proportion of the magnitude of the measurements themselves.

To determine the precision of the measurements, the 95% limits of agreement, calculated as ± 1.96 times the standard deviations of the differences were utilised.

## Results

Twelve participants (mean ± SD, 67.7 ± 9.0 years; 9 females) completed a total of 24 sleep studies with 21 concurrent actigraphy recordings.

Table 1 shows the 2-night pooled objective sleep measurements obtained from both actigraphy and PSG. While actigraphy demonstrated strong intraclass correlations with PSG for TST (ICC = 0.79, *p* = 0.001) and SE (ICC = 0.85, *p* < 0.001), it tended to overestimate these parameters ([report how much on average]). Weak intraclass correlations were observed for TIB (ICC = 0.30, *p* = 0.433), WASO (ICC = 0.33, *p* = 0.386), and SOL (ICC = 0.32, *p* = 0.056). Additionally, actigraphy significantly underestimated the number of awakenings (ICC = 0.45, *p* = 0.021) by about half on average.

**Table 1:** Overall objective reliability of sleep measurements shown by PSG sleep study report compared to geneactiv actigraphy

Variable	PSG	Actigraphy	ICC	*P*-value
N	21	21		
Time in bed (min)	545.7 ± 40.5	525.0 ± 53.1	0.30	0.433
Total sleep time (min)	375.6 ± 69.6	423.2 ± 63.5	0.79	0.001*
Wake after sleep onset (min)	128.9 ± 48.4	85.9 ± 46.3	0.33	0.386
Sleep efficiency (%)	68.7 ± 10.8	80.7 ± 9.7	0.85	< 0.001*
Sleep onset latency (min)	25.0 [15.5, 78.5]	1.0 [0.0, 10.0]	0.32	0.056
Number of awakenings	32.6 ± 12.2	16.0 ± 4.0	0.45	0.021*

Normally distributed data are reported as mean ± SD. Non-normally distributed data are reported as median [25% Percentile, 75% Percentile]. *Indicates significant correlation between nights for the same measurement device

**Fig. 1:**
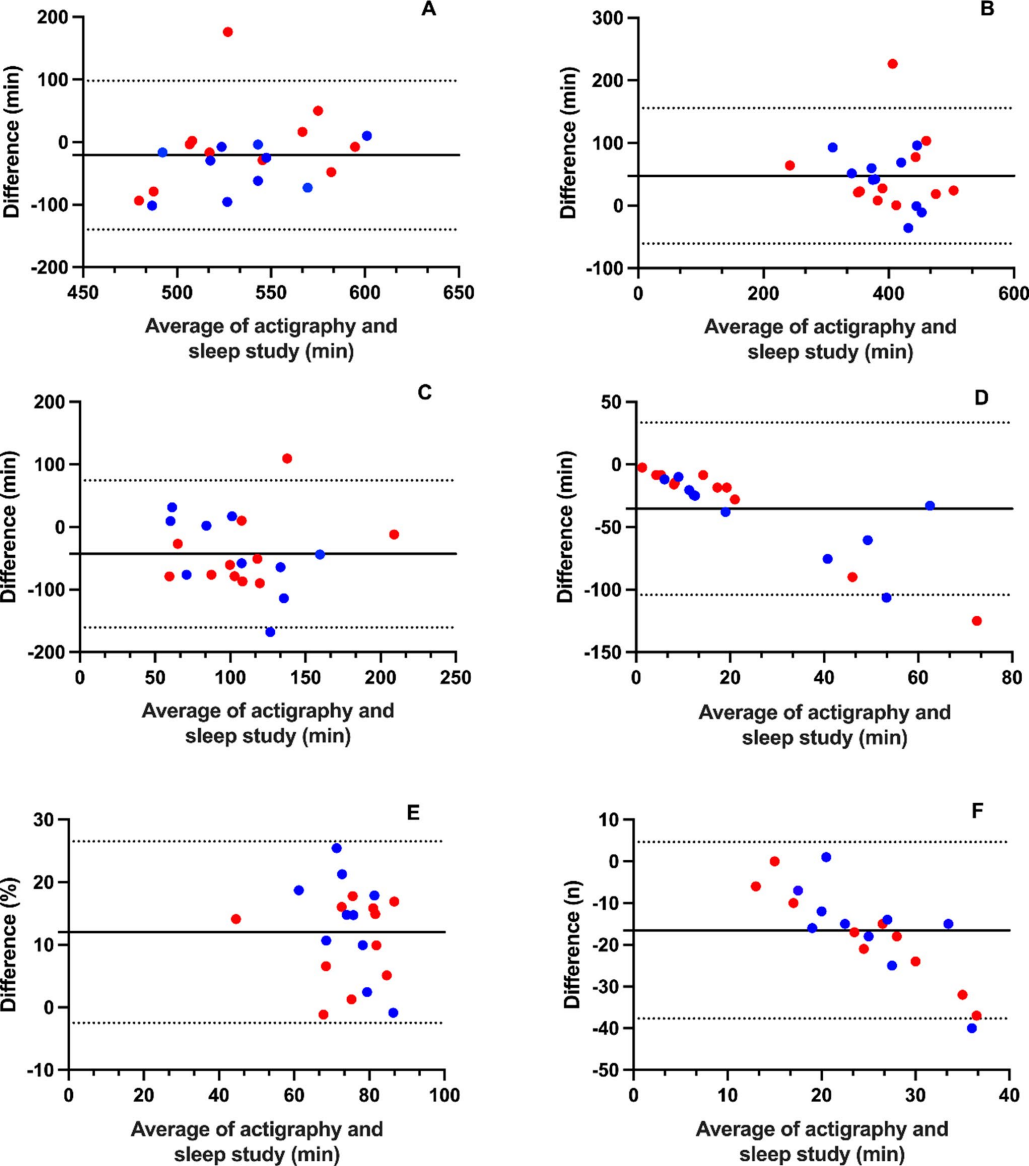
Bland-Altman graphs of sleep parameter measurements between actigraphy and Sleep Study (PSG - actigraphy)

The Bland-Altman plots (Fig. 1) illustrated significant proportional bias in SOL and the number of awakenings, suggesting that the magnitude of measurement discrepancies varies depending on the specific sleep parameter.

The within night analyses (night 1 and night 2) showed that the agreement between PSG and GeneActiv actigraphy measurements varies across different sleep parameters (Table 2). For both nights, actigraphy overestimated TST and SE, with ICCs of 0.80 (*p* = 0.020) and 0.67 (*p* = 0.200) for Night 1, and 0.70 (*p* = 0.028) and 0.77 (*p* = 0.055) for Night 2, respectively. In contrast, the ICCs for TIB were moderate to low (0.52 for Night 1 and 0.13 for Night 2), with non-significant p-values (*p* = 0.118 and *p* = 0.846, respectively), indicating less agreement. The correlation for WASO was particularly low for both nights. Similarly, SOL showed poor agreement for Night 1 and Night 2. Lastly, the Number of Awakenings has weak correlations for both nights (ICC = 0.24, *p* = 0.582 for Night 1; ICC = 0.28, *p* = 0.004 for Night 2).

**Table 2:** Reliability of average of objective sleep measurements between nights for each method of measurement

	Night 1		ICC	*p*-value	Night 2		ICC	*p*-value
	PSG	Actigraphy			PSG	Actigraphy		
Time in bed (min)	551.7 ± 36.0	511.3 ± 44.3	0.52	0.118	540.3 ± 45.3	537.3 ± 59.3	0.13	0.846
Total sleep time (min)	376.7 ± 62.8	417.1 ± 42.2	0.80	0.020*	374.6 ± 78.3	428.6 ± 79.8	0.70	0.028*
WASO (min)	127.2 ± 58.7	80.9 ± 30.8	0.15	0.782	130.5 ± 39.7	90.39 ± 58.15	0.37	0.356
Sleep efficiency (%)	68.2 ± 10.0	81.7 ± 5.7	0.67	0.200	69.22 ± 11.93	79.9 ± 12.5	0.77	0.055
Sleep onset latency (mins)	31.5 [21.5, 79.0]	0.5 [0.0, 4.0]	0.47	0.241	18.5 [9.5, 35]	1.0 [0, 10]	0.10	0.248
Number of awakenings (n)	32.9 ± 10.8	16.8 ± 4.3	0.24	0.582	32.3 ± 13.9	15.4 ± 3.5	0.28	0.004*

Normally distributed data are reported as mean ± SD. Non-normally distributed data are reported as median [25% Percentile, 75% Percentile]. *Indicates significant correlation between nights for the same measurement device

## Discussion

This study evaluated the reliability of the GeneActiv actigraphy device in measuring sleep parameters compared to PSG measurements in older adults with self-reported sleep disturbance. Our findings expand upon prior research by demonstrating that actigraphy performance remains relatively stable across nights, suggesting that night-to-night variability does not significantly affect the reliability of GeneActiv sleep estimates in older adults. This contrasts with earlier research where first-night effects have been a major concern in PSG-based sleep studies. Our results demonstrated that while the GeneActiv device showed strong agreement with PSG for total sleep time (TST) and sleep efficiency (SE), significant discrepancies were observed for sleep onset latency (SOL) and wake after sleep onset (WASO). The GeneActiv actigraphy device exhibited a tendency, consistent with other actigraphy devices [5, 6, 11], to overestimate total sleep time and underestimate sleep onset latency and wake after sleep onset when compared to PSG measurements. In clinical settings, this could lead to underestimation of sleep fragmentation and delayed sleep onset, potentially affecting the management of sleep disorders in older adults.

We used Bland-Altman plots to visualise the agreement between the two methods and showed notable limitations. Firstly, the wide limits of agreement (LOA) observed in Bland-Altman plots highlight a substantial variability in the discrepancies between actigraphy and PSG measurements. For example, the mean difference in total sleep time (TST) of approximately 30 min, coupled with variability of up to +/– 100 min, underscores the potential for significant measurement errors. Such variability may impact clinical interpretations and patient management. To minimise the potential for first-night effects, where participants’ sleep patterns may be altered due to the novelty of the sleep lab environment, The study included two habituation nights before the testing night. As a result, the data analysed were from the third night, when participants were likely more accustomed to the sleep lab conditions. This approach helps ensure that the sleep patterns observed are more representative of typical sleep.

Several factors could have influenced our findings. The small sample size may limit the generalisability of the results, particularly regarding the variability across different sleep parameters. Additionally, comorbid conditions common in older adults could have introduced variability in sleep patterns. Future research should include larger, more diverse samples and consider assessing the impact of specific comorbidities. Improving algorithms to account for subtle physiological changes, such as heart rate variability or respiratory signals, could enhance the accuracy of GeneActiv measurements for parameters like SOL and WASO. Future studies should also include diverse age groups and clinical populations to validate the device’s performance across different demographics and sleep disorders.

In conclusion, While GeneActiv shows promise for estimating TST and SE, it cannot fully replace PSG for detailed sleep assessments. Clinicians should use actigraphy as a supplementary tool, particularly for monitoring broad sleep trends, while recognizing its limitations in capturing precise metrics such as WASO and SOL. Researchers and clinicians can leverage this tool’s affordability and additional functionalities for various sleep monitoring applications. However, the significant variability in some measurements, particularly sleep onset latency and wake after sleep onset, indicates that caution should be exercised when interpreting actigraphy data. Further advancements in wearable technology and algorithm refinement may continue to bridge the gap between actigraphy and PSG, ultimately enhancing the field of sleep research and clinical practice.

## Data Availability

Data used in this study will be made available on reasonable request and approval of the Sydney Local Health District Human Research Ethics Committee.
